# Chromosomal copy number analysis on chorionic villus samples from early spontaneous miscarriages by high throughput genetic technology

**DOI:** 10.1186/s13039-015-0210-z

**Published:** 2016-01-26

**Authors:** Jiandong Shen, Wei Wu, Chao Gao, Humphrey Ochin, Dianyun Qu, Jiazi Xie, Li Gao, Yadong Zhou, Yugui Cui, Jiayin Liu

**Affiliations:** Department of Reproductive Medicine, The First Affiliated Hospital of Nanjing Medical University, State Key Laboratory of Reproductive Medicine, Nanjing, 210029 China

**Keywords:** Array comparative genomic hybridization, Next generation sequencing, Spontaneous miscarriage, Chorionic villus samples, Chromosome

## Abstract

**Background:**

About 10 –15 % of all clinically recognized pregnancies result in spontaneous miscarriages, and chromosomal abnormalities are the most common reason. The conventional karyotyping on chorionic villus samples (CVSs) is limited by cell culture and its resolution. This study aimed at evaluating the efficiency of the application of high throughput genetic technology, including array comparative genomic hybridization (array CGH) and next generation sequencing (NGS) on the chromosomal copy number analysis of CVSs from early spontaneous miscarriages.

**Results:**

Four hundred and thirty-six CVSs from early spontaneous abortion were collected. Genomic DNA was extracted using a routine method, and the chromosomal copy number variants (CNVs) were analyzed by array CGH and NGS. Two hundred and twenty-five samples (51.6 %) with abnormal chromosomes were identified among 436 samples, of which 188 samples (41.3 %) were aneuploidy, 23 samples (5.3 %) were segmental deletion and/or duplication cases, and 14 samples (3.2 %) were triploid. Two of the three cases with small segmental deletion and duplication were validated to be transferred from their fathers who were carriers of submicroscopic reciprocal translocation.

**Conclusion:**

A high chromosomal abnormality detection rate on CVSs from early spontaneous miscarriage was achieved by array CGH and NGS. Specifically, the detection of submicroscopic recombination, which is sometimes missed by conventional karyotyping, was important for genetic counseling for the couples that suffered from recurrent miscarriages.

## Background

Miscarriage is the most common complication of pregnancy. About 10 –15 % of all clinically recognized pregnancies result in spontaneous miscarriage [1]. Chromosomal abnormalities account for ~45 % of early spontaneous miscarriages [2]. G-banding karyotyping is a traditional method of chromosomal analysis, and it plays an important role in investigating the reason for spontaneous abortion. However, G-banding karyotyping is hampered by poor chromosome preparations, culture failure, and maternal cell contamination. Molecular karyotype approaches, such as multiplex fluorescence *in situ* hybridization (mFISH), multiplex ligation-dependent probe amplification (MLPA), and quantitative real-time polymerase chain reaction (qPCR), have overcome some disadvantages inherent to conventional cytogenetic techniques. However, they are criticized for their restricted resolutions and/or limited coverage on the whole genome [3–5]. In this study, we apply array comparative genomic hybridization (array CGH) and next generation sequencing (NGS) technology to detect chromosomal abnormalities on chorionic villus samples (CVSs) from women who had early spontaneous miscarriages.

## Methods

### Samples collection and DNA extraction

The study was approved by Institutional Review Board of the First Affiliated Hospital of Nanjing Medical University. With informed consent, four hundred and thirty-six CVSs from women who had spontaneous miscarriages were collected, and all the miscarriages occurred between 5 to 12 weeks in gestational age. Each sample was rinsed in normal saline solution three times. Then 10 mg of the tissue was submitted to extract genomic DNA using a DNA extraction kit (Tiangen, China).

### Chromosomal copy number analysis by array CGH

Genomic DNA samples were fluorescently labelled and competitively hybridized to CytoChip Focus Constitutional microarrays (Illumina, USA) with a normal male control gDNA in an array CGH experiment format. A laser scanner InnoScan w710AL (Innopsys, France) was used to excite the hybridized fluorophores and read and store the resulting images of the hybridization. Scanned images were then analyzed and quantified by an algorithm with fixed settings in BlueFuse Multi Software (Illumina, USA) (available protocol at www.cytochip.com).

### Chromosomal copy number analysis by NGS and validation

Whole genome sequencing by NGS technology was performed on an Ion torrent PGM (ThermoFisher, USA) platform according to the standard protocol (protocol available at https://ioncommunity.thermofisher.com/community/protocols-home). Genomic DNA from CVSs was sheared to 250–300 bp fragments using Ion Shear Plus Reagents Kit (ThermoFisher, USA). Ion Torrent barcoded libraries were made using Ion Plus Fragment Library Kit (ThermoFisher, USA). Ion PGM Template OT2 200 Kit (ThermoFisher, USA) was used for template amplification and enrichment of target sequence. Ion Sphere Particles (ISPs) were recovered and template-positive ISPs were enriched using an Ion OneTouch ES (ThermoFisher, USA). Sequencing was performed using an Ion PGM Sequencing 200 Kit v2 (ThermoFisher, USA) on ‘318’ sequencing chip for a total of 500 nucleotide flows, yielding average read lengths of 220–230 bp. Ten DNA samples were pooled together and labeled with different barcodes on ‘318’ chip. The average whole genomic sequence depth was ~0.02×, and the average read number was ~500 K. The primary sequencing BAM data were submitted to the Celloud cloud server (available at http://www.celloud.org/), which was offered by a third-party company (JBRH, China), in order to analyze the chromosomal copy number variants (CNVs). The pipeline of the data analysis was done according to the previous report [6]. Before using NGS to detect chromosomal CNVs routinely, validation work was performed. Ten CVSs with different types known of chromosomal abnormalities, which were confirmed by array CGH, were submitted to sequence blindly. Subsequently the consistency of the results between NGS and array CGH were analyzed.

### G-banding karyotyping

Lymphocytes that were isolated from the patients were cultured and harvested after stimulation with phytohemagglutinin for 72 h. Metaphase chromosomes were prepared according to standard cytogenetic protocols. Karyotypes were described according to the International System for Human Cytogenetic Nomenclature 2013 (ISCN 2013).

### Parental origin analysis of chromosomal aberration by FISH

FISH was performed on metaphase chromosomes of the lymphocytes using telomere probes (Vysis, IL) according to the previous protocol [7]. Lymphocytes were cultured and harvested after stimulation with phytohemagglutinin for 72 h, and metaphase chromosomes were fixed on slides. After degeneration at 78 °C for 5 min, probes with fluorescence labeling were hybridized to the chromosomes on the slides at 37 °C for 16 h. The slides were washed in 2 × SSC (Sigma, USA) and dyed with DAPI (Vysis, IL). The signals under a fluorescence microscope were observed (Leica, GER).

## Results

**Fig. 1:**
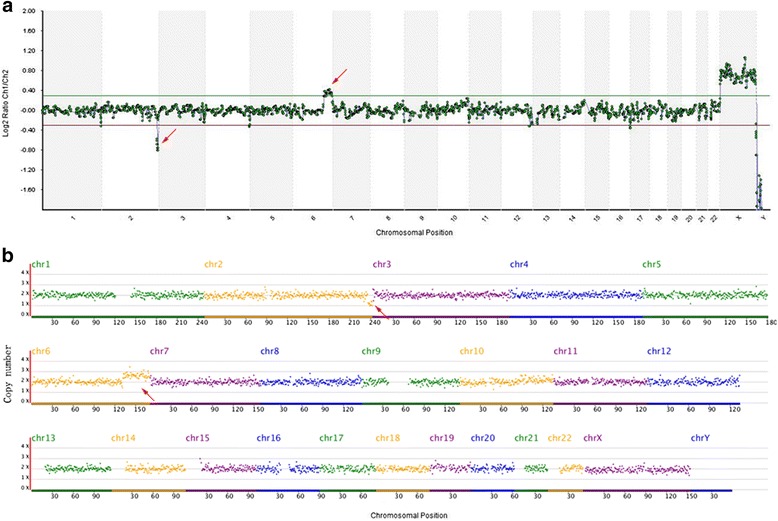
Comparison of chromosomal copy number analysis by NGS and array CGH. The red arrow indicates del(2q37.3-qter)(4.6 M) and dup(6q23.2-qter)(36.1 M) in sample C0012. Chart **a** shows the array CGH results, and **b** shows the NGS results

The NGS results of the 10 samples were completely consistent with those of array CGH (see Table 1 and Fig. 1); therefore, more samples were submitted for testing using the NGS method. A total of 436 samples were tested, 256 cases of which were tested by array CGH, and 180 cases were tested by NGS. Because detection coverage was theoretically consistent based on array CGH and NGS, we calculated the detection results by using the two methods together. Two hundred and twenty-five cases were found to have abnormal chromosomes, which accounted for 51.6 % of all the cases. There were 188 (43.1 %) cases with aneuploidy, 23 (5.3 %) cases with chromosomal segmental duplication and/or deletion, and 14 (3.2 %) cases with polyploidy (see Table 2). A total of 110 female samples and 101 male samples were found in the normal samples.

**Table 1:** Validation of copy number analysis by NGS

NO	Results by array CGH	Consistency with NGS
C0005	+2;XY	Yes
C0003	+16;XX	Yes
C0015	+21;XX	Yes
C0029	-X	Yes
C0142	+15(Mosaic);XY	Yes
C0021	69, XXY	Yes
C0123	69, XYY	Yes
C0012	-(2q37.3-qter)(4.6 M), +(6q23.2-qter)(36.1 M);XX	Yes
C0146	+(9q34.11-qter)(9.2 M), −(14q32.13-qter)(14.8 M);XX	Yes
C0179	Euploid;XX	Yes

**Table 2:** Summary of chromosomal copy number analysis of CVSs

Method	Maternal age	Chromosomal abnormality				Euploidy	Total
		Aneuploidy	Dup/Del	Polyploidy^a^	Total		
Array CGH	30.8 ± 4.3	115(44.9 %)	7(2.7 %)	9(3.5 %)	131(51.2 %)	125(48.8 %)	256
NGS	31.1 ± 4.8	73(40.6 %)	16^b^(8.9 %)	5^c^(2.8 %)	94(52.2 %)	86(47.8 %)	180
Total	30.9 ± 4.5	188(43.1 %)	23(5.3 %)	14(3.2 %)	225(51.6 %)	211(48.4 %)	436

^a^array CGH and NGS could identify some polyploidy such as 69 XXY and 69 XYY, but could not find 69 XXX, which was no sex chromosomal segregation. ^b^two of them combined with aneuploidy;^c^one of them combined with segmental duplication and tetrasomy

**Fig. 2:**
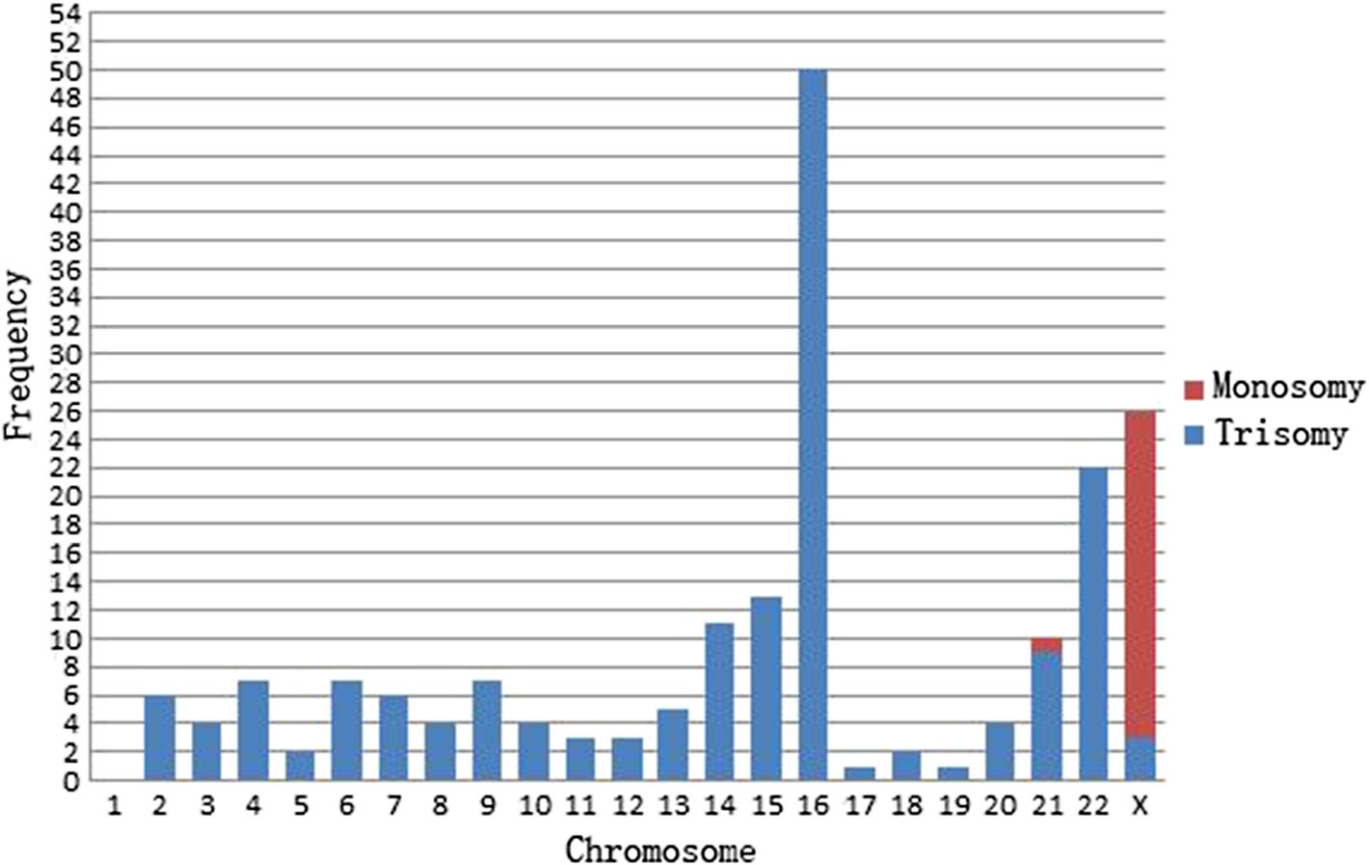
The frequency of each chromosomal aneuploidy

As observed in this study, aneuploidy was involved in almost all the chromosomes, except chromosome 1, and trisomy was the most common, especially in chromosome 16. Monosomy was mainly found in sex chromosomes except for one case with monosomy 21. Some aneuploidy samples involved two chromosomes. The total chromosomal numerical abnormality frequency was 198, for all the chromosomes except chromosome 1. Trisomy 16 was the most common in 50/198 (25.3 %), followed by chromosomes X, 22, 15, 14, and 21 (see Fig. 2).

**Fig. 3:**
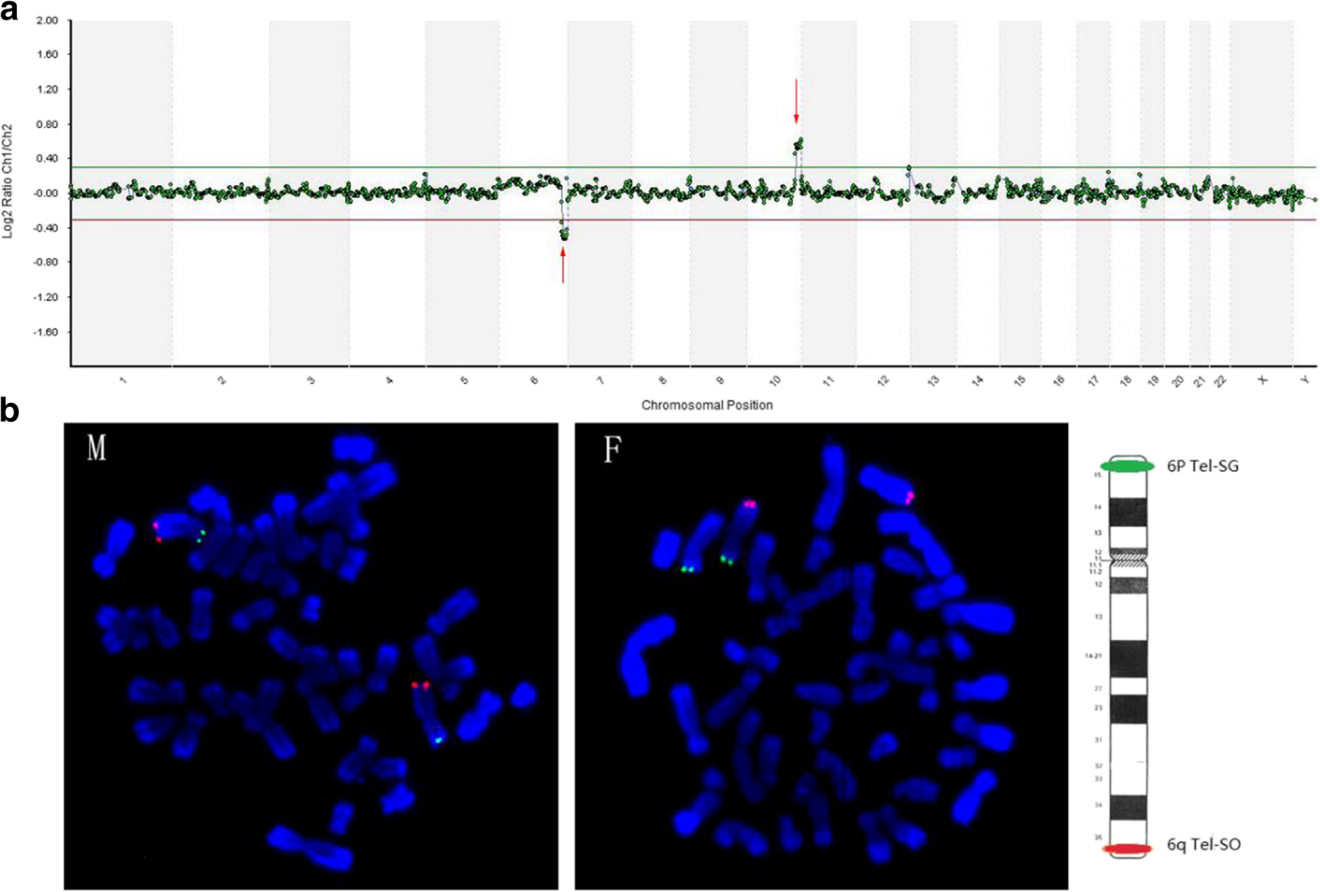
Parental origin analysis of submicroscopic segmental duplication and deletion. Sample C0044: Chart **a** shows the result of array CGH indicating del(6q25.3-qter) (12.5 M) and dup(10q26.11-qter) (13.8 M). Chart **b** shows the results of FISH labeling 6p with green signal and 6q with orange signal, indicating normal signals with mother(M) and reciprocal translocation with father(F)

Segmental deletion and/or duplication was found in 23/436 (5.3 %) (see Table 3). Seventeen couples’ karyotypes were available, and fifteen couples were reported as having normal karyotype, except for 2 reciprocal translocation carriers. Three couples with normal karyotype were submitted for FISH analysis, and two of them were identified to be submicroscopic reciprocal balanced translocation (see Table 4 and Fig. 3).

**Table 3:** Parents karyotype analysis of the CVSs with segmental copy number variants

No	Copy number variants for CVSs	Parents karyotype
C0012	-(2q37.3-qter)(4.6 M); +(6q23.2-qter)(36.1 M)	Normal
C0044	-(6q25.3-qter)(12.5 M); +(10q26.11-qter)(13.8 M)	*Normal*
C0146	+(9q34.11-qter)(9.2 M); −(14q32.13-qter)(14.8 M)	*Normal*
C0391	-(1p36.21-pter)(12.36 M)	Normal
C0001	-(18p11-pter)(14.0 M); +(18q11-qter)(59.4 M)	Normal
C0052	+(17q21.31-qter)(38.9 M)	Normal
C0227	-(8p12-pter)(28.8 M); +(8q24.3-qter)(4.9 M)	Normal
C0361	+(5q13.2-qter)(106.42 M); −(15q26.1-qter)(11.35 M)	Normal
C0376	-(5p15.1-pter)(16.6 M); +(9q21.32-qter)(56.6 M)	Normal
C0403	-(5p14.1-pter)(25.68 M); +(19q13.33-qter)(8.35 M)	Normal
C0407	+(2q12.1-q33.1)(93.02 M)	Normal
C0419	+19(q13.33-qter)(7.56 M)	Normal
C0420	+(11q23.3-pter)(111.15 M); −(22q11.1-q11.21)(3.92 M)	Normal
C0432	+(9p21.3-pter)(21.71 M); −3(q28-qter)(8.3 M)	Normal
C0439	-(1p36.21-pter)(13.69 M); +19	Normal
C0404	+(5p13.33-pter)(31.09 M); −(10q24.32-qter)(29.19 M)	Paternal reciprocal translocation carrier
C0193	+(2p24.3-pter)(15.11 M); −(13q22.1-qter)(38.47 M)	Paternal reciprocal translocation carrier
C0063	+(16p11.2-qter)(56.4 M)	Loss to Follow-up
C0195	-(8q24.13-qter)(19.83 M); +(11q23.3-qter)(17.22 M)	Loss to Follow-up
C0294	+(11q22.3-q24.2)(22.08 Mb); −(11q24.2-qter)(7.12 Mb)	Loss to Follow-up
C0358	-(13q21.31-qter)(48.84 Mb)	Loss to Follow-up
C0385	+14(q11.2-q12)(8.67 M); −X	Loss to Follow-up
C0389	-(18p11.21-pter)(14.08 Mb); +(18p11.21-qter)(59.8 Mb); +(19q12-qter)(27.58 Mb)	Loss to Follow-up

**Table 4:** Origin analysis of 3 cases with small-size segmental imbalances by FISH

No.	Copy number variants	FISH test			Parental origin
		Probe^a^	Paternal	Maternal	
C0012	-(2q37.3-qter)(4.6 M);	6p SG; 6q SO	Normal	Normal	De novo
	+(6q23.2-qter)(36.1 M)				
C0044	-(6q25.3-qter)(12.5 M);	6p SG; 6q SO	Carrier	Normal	Paternal
	+(10q26.11-qter)(13.8 M)				
C0146	+(9q34.11-qter)(9.2 M);	14q SO	Carrier	Normal	Paternal
	-(14q32.13-qter)(14.8 M)				

^a^FISH test was performed on the metaphase of lymphocytes using telomeric probes. *SG* spectrum of green, *SO* spectrum of orange. Carrier, reciprocal balanced translocation carrier

## Discussion

Chromosomal abnormality is the main reason for first-trimester miscarriages. Conventional G-banding karyotyping is used as a golden standard method to detect chromosomal aneuploidy and imbalances. However, the overall detection failure rate was ~20 % [2, 4], and sometimes false negative outcomes resulted from the overgrowth of maternal cells in relation to fetal cells [5, 8, 9]. Array CGH is a rapid, automated, reliable, and high-resolution technique used to diagnose unbalanced chromosomal abnormalities in CVSs from miscarriage patients [10, 11]. Recently, NGS was validated as being able to reliably detect the CNVs in CVSs [12]. Array CGH and NGS are both high throughput genetic test platforms that had revolutionary impacts on traditional cytogenetics [13–15]. We validated the efficiency of chromosomal copy number analysis through the NGS method in our lab and summarized the total detection efficiency of array CGH and NGS methods on CVSs. Here, a total number of 436 CVSs from early miscarriages were analyzed by array CGH and NGS with a 100 % diagnosis rate. We achieved a 51.6 % detection rate.

The occurrence frequency of each aneuploid chromosome was analyzed in this study, which suggested that errors were involved in all chromosomes besides chromosome 1. However, limited chromosome probe panels were always used to analyze the prenatal samples [16], miscarriage samples [4, 17], and pre-implantation embryos [18, 19]. According to this study, the limited probe panel (Chr13,18,21,X,Y) can only detect 43/198 (21.7 %) aneuploidy in CVSs, and the probe panel (Chr13, 16, 18, 21, 22, X ,Y) can only detect 115/198 (58.1 %) as well. It is obvious that a limited chromosome analysis method for CVSs is not suitable because many positive samples would be ignored. This also reflects why pre-implantation genetic screening using mFISH was proved to have no benefit for improving in vivo fertilization outcomes [20].

It is generally acknowledged that the resolution of routine G-banding karyotyping is 5-10 Mb. However, when the chromosomal segmental imbalances were involved in atypical bands, or poor digestion and dyeing were taken place in the procedure of chromosome preparation, even more than 10 Mb segmental duplications or deletions were sometimes missed, such as samples C0044 and C0146 (see Table 4). Array CGH had been used to identify small-size CNVs on miscarriage samples [2]; however, most of the CNVs found in those studies had no parental origin analysis and no significant clinical value was concluded. In this study, segmental deletion and/or duplication was observed in 5.3 % of the samples by array CGH or NGS. We failed to analyze the origin of the segmental changes in all 15 of the couples with normal karyotypes. However, FISH was performed on three of them to investigate the parental origin, and two of them were proved to be hereditary from the paternal submicroscopic reciprocal balanced translocations. With exact chromosomal diagnosis, pre-implantation genetic diagnosis (PGD) was recommended to these couples [21]. Although we could not conclude the exact incidence of submicroscopic recombination in miscarriage couples with normal karyotypes, we emphasized that a clinician should be aware of submicroscopic reciprocal translocation in couples with recurrent miscarriages.

With consideration of identifying submicroscopic reciprocal translocation, high throughput genetic testing is recommended for analyzing the chromosomal copy number of CVSs from spontaneous abortions. Array CGH and NGS were robust in the detection of chromosomal CNVs, and 69 XXY and 69 XYY could be detected as well for the special segregation of sex chromosomes. However, the limitations also should be considered. NGS and array CGH cannot detect all polyploidies, such as 69 XXX, 92 XXXX, and 92 XXYY, as well as balanced translocations. In present study, more normal female samples were observed than normal male samples (110 *vs* 101), which was likely caused by confusion of 69 XXX and 46 XX. In the future, single nucleotide polymorphism analysis could be adopted in order to identify the polyploidies by NGS.

## Conclusions

In conclusion, a high chromosomal abnormality detection rate on CVSs from patients who had spontaneous miscarriages was achieved by array CGH and NGS. Particularly, submicroscopic recombination could be detected, which was important to genetic counseling. Array CGH and NGS are comprehensive, rapid, and high-resolution chromosomal copy number analysis methods.
